# Whole body‐diffusion weighted imaging for the assessment of treatment response in hairy cell leukaemia: A positive first step

**DOI:** 10.1002/jha2.158

**Published:** 2021-02-02

**Authors:** Ashwin Algudkar, Dima El‐Sharkawi, Matthew Cross, Nina Tunariu, Ayoma D Attygalle, Bhupinder Sharma

**Affiliations:** ^1^ The Royal Marsden Hospital London UK; ^2^ The Institute of Cancer Research London UK

**Keywords:** hairy cell leukaemia, imaging, MRI

## Abstract

We present the case of a patient diagnosed with hairy cell leukaemia (HCL) who subsequently developed biopsy confirmed bone lesions and underwent multiple lines of therapy. The reported incidence of bone lesions in HCL is 3%, and bony involvement can be associated with high tumour burden and aggressive disease. The commonly lytic bone lesions in HCL are difficult to accurately assess for response. Whole body diffusion weighted imaging (WB‐DWI) has been used clinically in multiple myeloma; we postulate clinical utility in HCL, where hypercellularity also applies. In our case, WB‐DWI appears to discriminate sites of active disease from bone response. We present the salient radiological and pathological images. To our knowledge, this is the first description of WB‐DWI in HCL; we support research of WB‐DWI in the staging, prognostication and response assessment of HCL.

A 73‐year‐old male diagnosed with hairy cell leukaemia (HCL) 27 years previously had multiple therapies including alpha‐interferon, several lines of purine analogues and splenectomy. He maintained adequate haematological response whilst having ongoing alpha‐interferon before sustaining a biopsy‐proven pathological T2 fracture.

Bone marrow trephine biopsy revealed dense infiltration of medium‐sized cells with well‐defined cell margins and widely spaced oval/indented nuclei (the typical *‘fried egg’* appearance, top left). *CD20*, *Annexin A1*, *Tartrate‐Resistant Acid Phosphatase*, *Cyclin D1*, *CD11c*, *CD123*, *CD25* and *DBA44* were expressed; *CD5* and *SOX11* were not. The *BRAF‐V600E* mutation was detected.

The patient received radiotherapy to T2 and vemurafenib/rituximab for 4 months. Despite blood count normalisation, bony pains persisted, and further vertebral collapses necessitated vertebroplasty. Positron emission tomography‐computed tomography (PET‐CT) showed multiple avid areas throughout the skeleton with some inactive lytic lesions. Bone marrow biopsy revealed persistent very heavy (>90%) HCL infiltration.

Whole body‐diffusion weighted imaging (WB‐DWI) showed PET‐avid lesions such as the L5 vertebral body (top right, red arrow) demonstrated restricted diffusion (high DWI signal [bottom left, red arrow] with corresponding lower apparent diffusion coefficient [ADC] signal [not shown]) and abnormal marrow signal (bottom right, red arrow) consistent with active disease. Other PET‐positive pelvic lesions (top right, blue arrow) demonstrated variable DWI and marrow signal (bottom left and bottom right respectively: blue arrows), likely reflecting treated disease. He received moxetumomab for ongoing bony involvement but no discernible response was seen on end‐of‐treatment PET‐CT.

Reported incidence of bone lesions in HCL is 3% with greater accuracy precluded by the absence of routine imaging. Small series suggest bony involvement can be associated with high tumour burden and
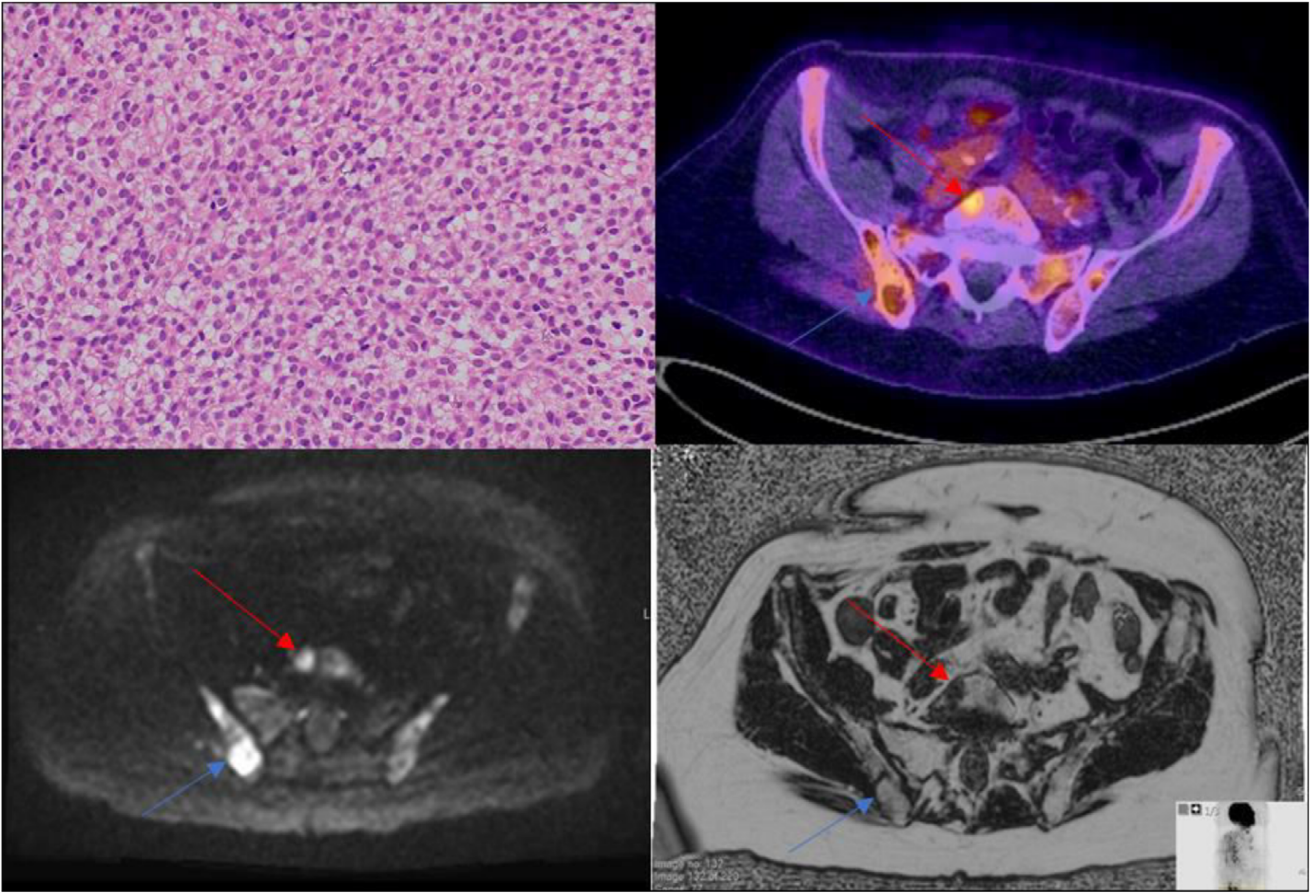
 aggressive disease. Bone lesions in HCL, commonly lytic, are difficult to accurately assess for response.

WB‐DWI measures Brownian motion of water protons over microscopic distances in the extracellular space; this is *‘restricted’* in highly cellular lesions. It has been used clinically in multiple myeloma; we postulate clinical utility in HCL, where hypercellularity also applies. WB‐DWI appears to discriminate sites of active disease from bone response. To our knowledge, this is the first description of WB‐DWI in HCL; we support research of WB‐DWI in HCL staging, prognostication and response assessment.

